# Effect of thyme-ivy syrup on antiviral immune response in patients with mild COVID-19: a prospective, open-label, randomized pilot study

**DOI:** 10.3389/fmed.2025.1672794

**Published:** 2025-10-22

**Authors:** Stephanie Dauth, Stephan M. G. Schäfer, Maximilian Klippstein, Ann C. Foldenauer, Christin Jonetzko, Tanja Roßmanith, Susanne Schiffmann, Maria J. G. T. Vehreschild, Gerd Geisslinger, Frank Behrens, Michaela Koehm

**Affiliations:** ^1^Fraunhofer Institute for Translational Medicine and Pharmacology ITMP, Frankfurt am Main, Germany; ^2^Fraunhofer Cluster of Excellence Immune-Mediated Diseases CIMD, Frankfurt am Main, Germany; ^3^Institute for Clinical Pharmacology, University Hospital Frankfurt, Goethe University Frankfurt, Frankfurt am Main, Germany; ^4^Department of Internal Medicine and Infectious Diseases, University Hospital Frankfurt, Goethe University Frankfurt, Frankfurt am Main, Germany; ^5^Department of Rheumatology, University Hospital Frankfurt, Goethe University Frankfurt, Frankfurt am Main, Germany

**Keywords:** thyme-ivy syrup, phytotherapy, mild COVID-19, inflammatory marker, blood cells, cough

## Abstract

**Background:**

Thyme-ivy syrup has anti-inflammatory activities and exerts beneficial effects on symptom relief and recovery time in patients with acute bronchitis. The objective of this exploratory study was to evaluate the effect of thyme-ivy syrup on immune response mediators in patients with mild COVID-19.

**Methods:**

This prospective, open-label, randomized, controlled, single-center pilot study (BroVID study; EudraCT 2021–003237-11) was conducted in adult outpatients with mild COVID-19, cough, and ≥1 additional symptom. Patients were randomly assigned to the thyme-ivy syrup group, which received three 5.4 mL doses of oral thyme-ivy syrup per day for 14 days, or a control group (no medication) in a 2:1 ratio. The primary objective was to demonstrate a clinically relevant treatment difference between the two groups in change from baseline to day 7 in blood parameters involved in the immune response, including interleukin (IL)-6, IL-10, tumor necrosis factor, and specific blood cell types. Secondary objectives included assessments of other blood parameters and time points, symptoms, and quality of life. Adverse events were reported at each study visit.

**Results:**

Twenty-one patients were enrolled in and completed the study (13 in the thyme-ivy syrup group, 8 in the control group). On day 7, numerically greater decreases in the thyme-ivy syrup group were observed for some mediators, including IL-10 (−17.7 vs. − 6.0 pg/mL; effect size 0.53) and IL-6 levels (−4.9 vs. − 0.9 pg/mL; effect size 0.87). Significant between-group differences were observed for changes in some parameters at day 4 (e.g., IL-10) and day 14. Baseline characteristics differed between the two groups, including a higher viral load, shorter duration of symptoms prior to study start, elevated levels of inflammatory markers, and more comorbidities in the thyme-ivy syrup group compared with control. Thyme-ivy syrup was well tolerated.

**Conclusion:**

The exploratory BroVID study identified treatment differences in immune response mediators with moderate to large effect sizes for thyme-ivy syrup vs. control in patients with mild COVID-19; these differences require validation in a larger confirmatory study. The findings may be subject to bias due to imbalanced baseline characteristics, the inter- and intra-individual heterogeneity of inflammatory markers, and the small sample size.

## 1 Introduction

The severe acute respiratory syndrome coronavirus 2 (SARS-CoV-2), which causes coronavirus disease-2019 (COVID-19), is not a localized respiratory infection, but a multisystem disease that affects numerous organs and systems throughout the body. The disease process is the result of a complex interplay between the immune system, inflammation, and blood clotting (1). A number of studies have identified significant alterations in both innate and adaptive immune system functioning in patients diagnosed with COVID-19 (2), including lymphocytopenia, modulation of neutrophils, and decreases in monocytes, eosinophils, and basophils; these changes have been associated with disease severity (2–5). Furthermore, the majority of patients with severe COVID-19 exhibit markedly elevated serum levels of pro-inflammatory cytokines, including interleukin (IL)-6, IL-1β, IL-2, IL-8, IL-10, interferon (IFN)-*α*, IFN-*γ*, and tumor necrosis factor (TNF) (2, 5, 6). Some pro-inflammatory markers have been correlated with disease severity, while others vary between early and late disease stages (7, 8). Changes in these markers may therefore provide insights into symptom reduction or disease recovery.

A fixed combination of thyme herb and ivy leaf fluid extracts has been employed as a therapeutic agent for the treatment of cough associated with infections or inflammations of the lower respiratory tract for over two decades (9). *In vitro* and *in vivo* studies have shown that the active compounds in this syrup manifest multiple anti-inflammatory effects (10, 11), and clinical studies have demonstrated the efficacy of this thyme-ivy syrup in providing symptom relief and reducing recovery time in patients with acute bronchitis and cough (9, 12). In addition to anti-inflammatory activity, the ingredients found in the thyme-ivy syrup are associated with smooth muscle relaxation and bronchial cleansing effects (13, 14). Thyme-ivy syrup is well-tolerated, has few side effects, and has shown effectiveness in children and adults (9, 12, 15).

In light of these findings, thyme-ivy syrup and other herbal remedies were incorporated into the German Respiratory Society’s guidelines for the symptomatic management of adults with cough (16). Phytotherapies are well accepted in Germany by both patients (17) and medical practitioners (18), and phytopharmaceutical use is associated with reductions in antibiotic prescriptions in patients with upper respiratory tract infections (19).

Preclinical data indicate that thyme-ivy syrup may modulate the initial antiviral immune response to SARS-CoV-2. Specifically, it inhibits the interaction between the SARS-CoV-2 viral protein spike and the angiotensin-converting enzyme 2 (ACE2) receptor, which serves as a viral entry port to cells, and upregulates the secretion of IFN-*γ* in virus-stimulated peripheral blood mononuclear cells *in vitro* (11). In the presence of the SARS-CoV-2 spike protein, thyme-ivy syrup significantly increases the release of defensin molecules (11), a family of cationic peptides that can inhibit viral infection *in vitro*, including SARS-CoV-2 infection (20).

The necessity for effective and secure treatments for COVID-19 continues to be a matter of significant concern. Given the established safety profile of thyme-ivy syrup (9, 12), this therapy was considered unlikely to induce severe adverse effects in patients with mild SARS-CoV-2 infection. In this exploratory pilot study, which was designed to provide information for a larger confirmatory study, we sought to assess the impact of thyme-ivy syrup on the immune response and on recovery from symptoms in patients with mild COVID-19.

## 2 Methods

### 2.1 Study design

The thyme-ivy syrup in COVID-19 (BroVID) study was a single-center, prospective, open-label, randomized, two-arm pilot trial conducted at the Fraunhofer Institute for Translational Medicine and Pharmacology ITMP in Frankfurt am Main, Germany. The objective was to evaluate clinically relevant treatment differences between the thyme-ivy syrup and control groups in blood parameters involved in the immune response that would be informative for a subsequent confirmatory trial. Additionally, the study aimed to evaluate the effect of thyme-ivy syrup on the recovery of ambulatory adult patients with mild COVID-19. The study was registered before study initiation at EudraCT (2021–003237-11) and subsequently at clinicaltrials.gov (NCT05276375). The study protocol was approved by the ethics committee of Goethe University (reference number EK No 2021–307-AMG). All patients provided written informed consent prior to initiation of protocol procedures.

**Figure 1:**
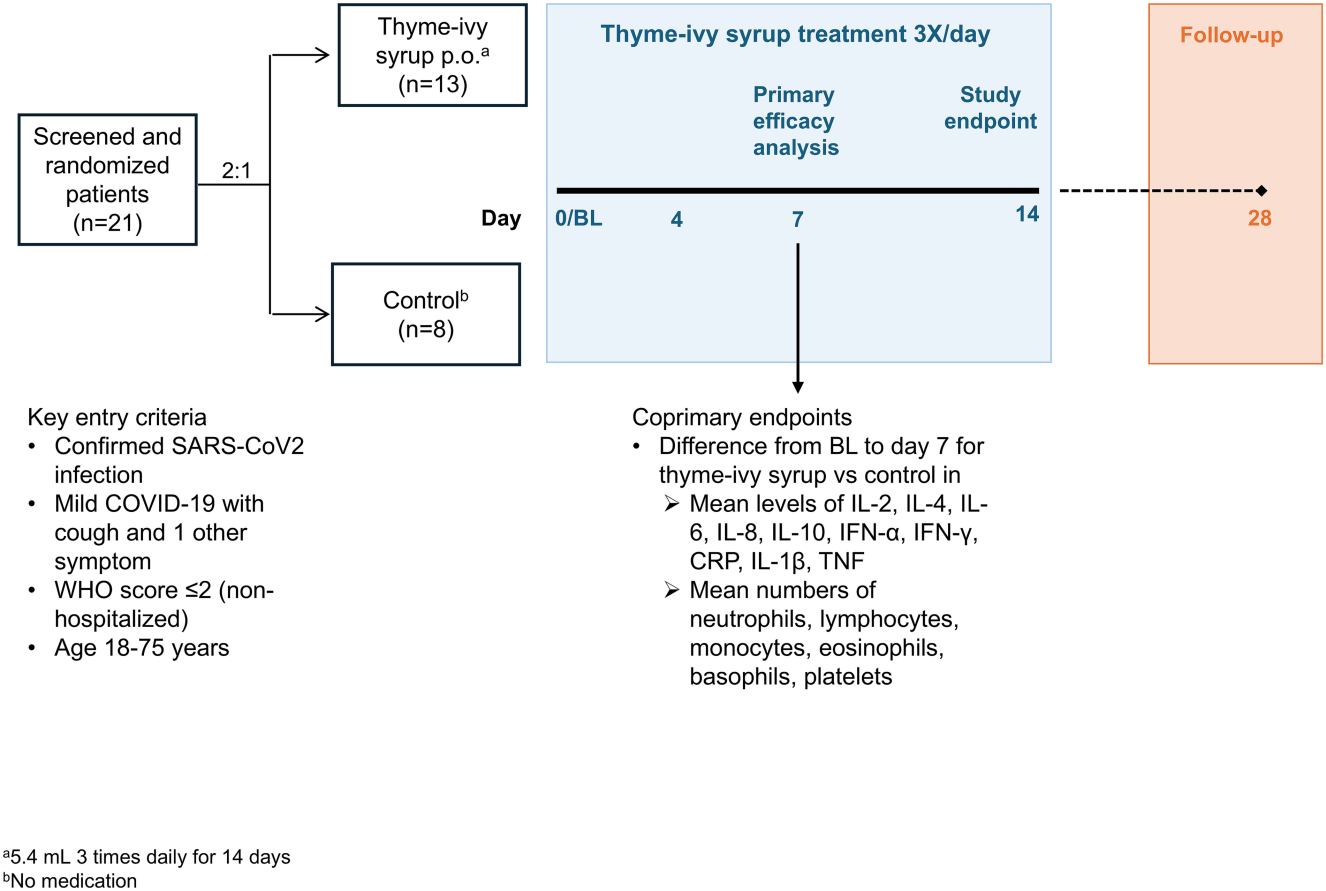
BroVID study design. All enrolled patients completed the 28-day study. BL, baseline; COVID-19, coronavirus disease-2019; CRP, C-reactive protein; IFN, interferon; IL, interleukin; SARS-CoV-2, severe acute respiratory syndrome coronavirus 2; TNF, tumor necrosis factor.

The BroVID study was conducted between March 17, 2022 (first patient visit) and May 31, 2023 (last patient visit). It commenced with a pre-screening telephone call, which was initiated by interested patients who had seen flyers concerning the study at COVID-19 test centers, general medical practices, or other healthcare locations. The pre-screening visit was followed by a baseline (BL) visit (day 1), which constituted the start of the treatment period (Figure 1). At the BL visit, patients were randomly assigned to either the thyme-ivy syrup (Bionorica SE, Neumarkt, Germany) group or the control group in a 2:1 ratio by a computer-generated randomization procedure. The patients in the thyme-ivy syrup group received a bottle of thyme-ivy syrup (provided by Bionorica) as a regular product and were instructed to take three doses of 5.4 mL thyme-ivy syrup per day for 14 days using the enclosed measuring cup as specified in the patient information leaflet (21). The patients in the control group did not receive any study medication, as this study was intended as a pilot study to provide information for a subsequent larger confirmatory trial.

Subsequent study visits were conducted at days 4, 7, and 14, and a follow-up video-conference or phone call was conducted at day 28 (Figure 1). Blood samples and throat swabs for a SARS-CoV-2 test by polymerase chain reaction (PCR) were obtained at BL and days 4, 7, and 14, and patients were evaluated by a physician or study nurse and asked about current medications and adverse events (AEs). Beginning at BL, patients were given copies of the Food and Drug Administration (FDA) symptom questionnaire (22) (described below) to be completed daily. Patients were provided a patient diary at BL and instructed to document the intensity of their most bothersome symptom (on a visual analogue scale [VAS] ranging from 0 [not bothersome at all] to 100 [very bothersome]) daily at home until day 14. All patients also recorded concomitant medications, and patients in the thyme-ivy syrup group recorded intake of thyme-ivy syrup. Open bottles of thyme-ivy syrup were collected by the study site at the end of the study as a further assessment of treatment compliance.

All concomitant medications that were taken during the treatment period were documented in the patient diary and reviewed at each study visit. Paracetamol (up to a maximum of 4,000 mg/ 24 h) was permitted, provided that it was discontinued for a period of 24 h prior to a scheduled official study visit. Concomitant medications listed in Supplementary Table S1 were prohibited until day 14, including cough and cold products and medications used to treat SARS-CoV-2 infection.

### 2.2 Study population

The full list of inclusion and exclusion criteria is presented in Supplementary Table S2. Briefly, the study included adult (≥ 18 to ≤ 75 years) patients with a SARS-CoV-2 infection confirmed by PCR ≤ 4 days before BL and symptom onset < 7 days before the screening/BL visit. Patients were required to have mild COVID-19 [World Health Organization (WHO) score ≤ 2] (23) with cough and at least one other symptom, such as sore throat, nasal congestion, or headache. Only ambulatory, non-hospitalized patients were included. Main exclusion criteria included a WHO score ≥ 3, other advanced or chronic lung diseases, BMI > 35 kg/m^2^ or body weight ≤ 43 kg, requirement for oxygen administration, regular intake of immunosuppressive medication (including steroids) or nonsteroidal anti-inflammatory drugs, and COVID-19 vaccination within the last 28 days or planned within the study period.

### 2.3 Objectives and outcomes

The primary efficacy objective was to demonstrate a clinically relevant treatment difference in change from BL in mean blood parameters involved in the immune response at day 7 between patients with mild SARS-CoV-2 infection who received thyme-ivy syrup and those who did not receive thyme-ivy syrup (control). Blood parameters specified for the primary endpoint included immune response mediators (IL-1β, IL-2, IL-4, IL-6, IL-8 [also known as chemokine CXC motif ligand 8 (CXCL8)], IL-10, IFN-*α*, IFN-*γ*, C-reactive protein [CRP], and TNF) and blood cells (basophils, eosinophils, lymphocytes, monocytes, neutrophils, and platelets). No analyses were conducted on changes in IL-1β, IL-2, IL-4, and IFN-α values due to too many values falling below the limit of quantification. Cytokine levels were assessed at Fraunhofer ITMP (Frankfurt, Germany) and hematology parameters and immune markers were assessed at a central laboratory (MLM Medical Labs, Mönchengladbach, Germany) using commercial assays.

Secondary efficacy objectives included assessment of the blood parameters evaluated for the primary objective at the other study visits (day 4 and day 14) and analysis of additional blood parameters, including ferritin, hemoglobin, immunoglobulin M (IgM), IgG, markers for tissue or organ damage (aspartate aminotransferase, alanine aminotransferase, and creatinine) and markers for thrombosis (activated partial thromboplastin time, prothrombin time, fibrinogen, anti-thrombin III activity), at all visits.

Improvements in symptoms were also evaluated, including symptomatic response based on the 2020 FDA symptom questionnaire in which 14 common symptoms are evaluated (22). Ten of the symptoms (stuffy or runny nose, sore throat, shortness of breath, cough, low energy/tiredness, muscle or body aches, headache, chills or shivering, feeling hot or feverish, and nausea) are evaluated on a scale from 0 (none) to 3 (severe) based on worst severity over the past 24 h, two (vomiting and diarrhea) are evaluated on a scale from 0 (none) to 3 (5 or more times in the last 24 h), and two (sense of smell, sense of taste) are evaluated on a scale from 0 (same as usual) to 2 (no sense of smell/taste).

Additional assessments included quality of life evaluations based on two patient-reported global impression items as recommended by the FDA (22). Specifically, patients were asked about their “return to usual health” and “return to usual activity” (prior to their COVID-19 diagnosis) in the past 24 h, with answers recorded as “Yes” or “No.” SARS-CoV-2 infection was measured at BL, day 4, day 7, and day 14 using PCR. The cycle threshold (Ct) value, which refers to the number of cycles needed to replicate sufficient DNA for detection, was evaluated at BL.

The WHO Clinical Progression Scale, which was designed to provide a common outcome measure set for COVID-19 clinical research (23), was used to document COVID-19 severity and progression over time. The scale provides a score from 0 to 10 defining different severity states of the disease: uninfected (0), ambulatory mild disease (1–3), hospitalized: moderate disease (4, 5), hospitalized: severe disease (6–9), and dead (10) (23).

Safety, as determined by AE reports, was evaluated at each study visit and at the 28-day follow-up.

### 2.4 Statistical analysis

Pilot studies are exploratory in nature and are designed to test the feasibility of clinical trials and to estimate outcome parameters and their variability on a smaller scale (24). Our goal was to recruit approximately 10% of the population required for a confirmatory clinical trial (25–27). We calculated that a confirmatory trial with a single endpoint and a low to moderate effect size of approximately Cohen’s d = 0.35 would require a sample size of 292 patients to achieve 80% power in demonstrating superiority of thyme-ivy syrup in comparison to the control group with respect to the primary endpoints (*α* = 5%, two-sided t-test, G*Power 3.1.9.6). For this pilot study, we estimated that a sample size of 30 patients randomized 2:1 (thyme-ivy syrup group:control group), representing approximately 10% of patients for a confirmatory trial, would be sufficient to assess the preliminary effects of thyme-ivy syrup on the immune response and symptoms in patients with mild COVID-19.

Analyses were conducted on the full analysis set (FAS), defined as all patients who were randomized and received study medication at least once. The FAS was identical to the per protocol set. Endpoints were assessed using descriptive statistics and exploratively using longitudinal mixed models. Data are given as mean values and standard deviation (SD) unless specified otherwise. Although the study was not powered for confirmatory statistical analyses, exploratory statistical tests were performed at a 5% significance level.

The primary analysis entailed comparisons of changes from BL to day 7 in mean blood parameter values between the thyme-ivy syrup and control groups. All missing values for the primary analysis were imputed by the last valid observation carried forward (LOCF) method. LOCF was acceptable, since the majority of blood parameters analyzed had no more than 1–2 patients with missing values at visit 3 (day 7). A linear mixed model with repeated measures (LMM) was fitted for each normally distributed laboratory value. BL blood parameter values, visit (BL, day 4, day 7, or day 14), treatment and visit-by-treatment interaction were included as covariates. Covariance structure was fitted using Akaike’s information criterion (AIC); autoregressive structure (rank 1), compound symmetry, and unstructured models were compared. LMM could only be fitted for basophils, CRP, CXCL8, eosinophils, IL-10, IL-6, lymphocytes, monocytes, neutrophils, platelets, and TNF. All of these, except for basophils, lymphocytes, monocytes and TNF, required a Box-Cox transformation to achieve normality of residuals (28). Wilcoxon tests were used to evaluate the treatment effect of non-normally distributed values. Secondary analyses utilized observed data with no data imputation.

Cohen’s effect sizes were added for immune response mediators and blood cells. The following effect sizes were considered: Cohen’s d for independent two-sided t-test (d = 0.2 indicates a small effect, d = 0.5 indicates a moderate effect, d ≥ 0.8 indicates a large effect) (29, 30). Because effect sizes may be biased by underlying baseline variables, effect sizes were interpreted with caution considering the results of the LMM. All analyses were conducted in SAS 9.4 (SAS Institute Inc., Cary, NC).

## 3 Results

Twenty-one patients were screened and all 21 were enrolled and randomized 2:1 to the thyme-ivy syrup (*n =* 13) or control (*n =* 8) groups (Figure 1). The study population was somewhat smaller than the originally intended 30 patient sample size due to the end of the pandemic and cessation of SARS-CoV-2 testing. There were no discontinuations during the study in either group; all 21 patients completed the study. Compliance with thyme-ivy syrup intake was quite high (99.6%). Three patients reported missing one dose each.

### 3.1 Baseline characteristics

The mean (standard deviation [SD]) age of the 21 patients in this study was 30.0 (8.9) (range, 19 to 48) years. Approximately half of the patients (11 [52.4%]) were female and 10 of the females were of child-bearing potential. None of the patients had a fever at BL and all had been vaccinated for SARS-CoV-2. All patients except one in the thyme-ivy syrup group had received three SARS-CoV-2 vaccinations.

The thyme-ivy syrup and control groups were well-matched with respect to BL age, BMI, ethnicity, and blood pressure (Table 1). The thyme-ivy syrup group had a lower proportion of females (46.2% vs. 62.5%) and fewer mean days from symptom start to the BL visit (3.1 vs. 4.6 days). Mean PCR Ct values based on the Ct value on day 0 when available was lower in the thyme-ivy syrup compared with the control group (23.9 vs. 27.4), indicating a higher viral load in the thyme-ivy syrup group (Table 1). Patients in the thyme-ivy syrup group also had a higher number of mean symptoms based on the 2020 FDA-recommended questionnaire of 14 COVID-19 symptoms (6.8 vs. 5.9), higher mean symptom severity (0.74 vs. 0.67), and a higher proportion of patients with cough as the most bothersome symptom (46.2% vs. 25.0%). The proportion of patients with hyposmia/anosmia was higher in the control group (62.5% vs. 53.9). Patients in the thyme-ivy syrup group had more concomitant diseases, including one patient with chronic bronchitis (Table 1).

**Table 1:** Baseline demographic characteristics, clinical characteristics, and concomitant diseases.

Characteristic	Thyme-ivy syrup (*n =* 13)	Control (*n =* 8)
Demographics		
Age, mean (SD) years	29.4 (8.0)	31.1 (10.7)
BMI, mean (SD) kg/m^2^	23.1 (3.7)	25.0 (4.8)
Female sex, *n* (%)	6 (46.2%)	5 (62.5%)
Ethnicity, *n* (%)		
Caucasian	12 (92.3%)	7 (87.5%)
Asian	0	1 (12.5%)
Other	1 (7.7%)	0
Blood pressure		
Systolic, mean (SD) mmHg	123.2 (12.6)	120.5 (13.2)
Diastolic, mean (SD) mmHg	73.8 (9.9)	69.8 (12.0)
Symptom duration		
Number of days from symptom start to BL visit, mean (SD)	3.1 (1.4)	4.6 (1.1)
SARS-CoV-2 PCR data		
Smallest Ct value (at any time between day −3 and day 0)	23.9 (3.9)^a^	23.2 (4.0)
Days between PCR and BL	0.6 (0.9)^a^	1.1 (1.2)
Ct value (day 0 preferred if available)	23.9 (4.0)^a^	27.4 (5.8)
Days between PCR and BL	0.6 (0.9)^a^	0.3 (0.7)
Symptoms (based on FDA questionnaire)^b^		
Number, mean (SD) [range]	6.8 (2.5) [4–11]	5.9 (1.1) [5–8]
Severity, mean (SD)	0.74 (0.27)	0.67 (0.23)
Hyposmia/anosmia, *n* (%)	7 (53.9%)	5 (62.5%)
Most bothersome symptom, *n* (%)		
Cough	6 (46.2%)	2 (25.0%)
Cough and sore throat	2 (15.4%)	0
Cough plus other symptoms	0	1 (12.5%)
Fatigue	2 (15.4%)	1 (12.5%)
Headache	0	2 (25.0%)
Muscle or body aches	1 (7.7%)	0
Shortness of breath, fatigue, and other symptoms	1 (7.7%)	0
Other symptoms (unspecified)	1 (7.7%)	2 (25.0%)
Concomitant diseases at screening/BL		
Psychiatric	3	0
Anxiety	1	0
Depression	1	0
Post-traumatic stress disorder	1	0
Metabolism and nutrition disorders	1	1
Fructose intolerance	1	0
Obesity	0	1
Respiratory, thoracic, and mediastinal disorders	1	0
Chronic bronchitis	1	0
Endocrine disorders	2	0
Hypothyroidism	2	0
Immune system disorders	1	2
Drug hypersensitivity	1	0
Food allergy	0	1
Hypersensitivity	0	1
Reproductive system and breast disorders	1	0
Dysmenorrhea	1	0
Skin and subcutaneous tissue disorders	1	0
Urticaria	1	0

^a^Two values were missing from these analyses. ^b^Based on 14 symptoms with a severity assessed on a scale of 0 (none) to 3 (severe or 5 or more times in the last 24 h) for all symptoms except hyposmia/anosmia (assessed from 0 [same as usual] to 2 [no sense of smell/taste]). BL, baseline; Ct, cycle threshold; PCR, polymerase chain reaction; SD, standard deviation.

### 3.2 Co-primary efficacy endpoints: changes from BL to day 7 in pre-specified immune mediator levels and blood cell counts

Mean BL values of pre-specified evaluable immune response mediators (IL-6, IL-8, IL-10, IFN-*γ*, CRP, and TNF) were higher in the thyme-ivy syrup group compared with the control group (Table 2). In particular, values for IL-6, IL-10, and IFN-γ were more than two-fold higher. In contrast, mean cell counts for pre-specified blood cells (basophils, eosinophils, lymphocytes, monocytes, neutrophils, and platelets) were similar between the two groups (Table 2). Except for platelets and lymphocytes, all blood parameters where a LMM could be fitted (basophils, CRP, CXCL8, eosinophils, IL-10, IL-6, monocytes, neutrophils, TNF) demonstrated a significant baseline effect (Supplementary Appendix 1). Visit effects were only significant for basophils, CRP, IL-10, monocytes, neutrophils, platelets and TNF (Supplementary Appendix 1).

**Table 2:** Primary efficacy analyses of difference in change from BL to day 7 in blood parameter values between the thyme-ivy syrup and control groups.

Blood parameter	Normal range	Thyme-ivy syrup (*n =* 13)				Control (*n =* 8)				*P*-value^a^	Effect size ^c^
		BL value	Day 7 value	Absolute change from BL to day 7	% change from BL to day 7	BL value	Day 7 value	Absolute change from BL to day 7	% change from BL to day 7		
Immune response mediators											
CRP, mg/L	F/M < 5.0	5.49 (4.27)	1.25 (1.24)	−4.24 (4.13)	−60.51 (34.82)	3.29 (2.25)	0.90 (0.81)	−2.39 (2.38)	−63.46 (35.52)	0.50	0.52
IFN-γ, median (min, max) pg/mL^b^	*	0.98 (0.09, 4.40)	0.14 (0.08, 0.35)	−0.83 (−4.22, 0.05)	−74.27 (−95.89, 27.95)	0.45 (0.14, 1.31)	0.21 (0.11, 0.28)	−0.29 (−1.04, 0.03)	−57.53 (−79.53, 11.46)	0.40^b^	0.54
IL-6, pg/mL	*	6.64 (5.80)	1.78 (1.02)	−4.86 (5.47)	−57.38 (31.11)	3.04 (1.93)	2.09 (1.07)	−0.94 (1.88)	−20.29 (48.84)	0.09	0.87
IL-8, pg/mL	*	6.11 (3.18)	4.22 (1.15)	−1.89 (3.04)	−19.50 (31.40)	4.72 (1.41)	4.14 (1.10)	−0.58 (1.45)	−7.73 (27.64)	0.66	0.51
IL-10, pg/mL	*	25.02 (27.31)	7.35 (2.66)	−17.67 (27.14)	−58.22 (19.84)	11.16 (6.39)	5.21 (2.57)	−5.95 (6.61)	−35.73 (64.17)	0.23	0.53
TNF, pg/mL	*	23.23 (6.95)	16.32 (4.65)	−6.91 (6.26)	−27.28 (18.64)	16.51 (2.78)	14.19 (2.11)	−2.31 (2.69)	−12.94 (13.32)	0.99	0.88
Blood cells per nL											
Basophils	F/M < 0.08	0.04 (0.02)	0.04 (0.01)	−0.0023 (0.017)	21.80 (89.12)	0.03 (0.01)	0.04 (0.01)	0.0075 (0.010)	28.75 (31.27)	0.55	−0.66
Eosinophils	F/M 0.03–0.44	0.18 (0.13)	0.15 (0.10)	−0.03 (0.12)	10.57 (67.21)	0.15 (0.17)	0.16 (0.10)	0.0075 (0.10)	73.87 (147.62)	0.27	−0.33
Lymphocytes	F 1.22–3.56 M 1.05–3.24	1.49 (0.36)	1.88 (0.56)	0.39 (0.36)	25.81 (24.39)	1.66 (0.24)	2.16 (0.61)	0.51 (0.49)	30.22 (27.92)	0.63	−0.29
Monocytes	F 0.25–0.85 M 0.26–0.87	0.61 (0.14)	0.44 (0.10)	−0.16 (0.18)	−21.92 (30.28)	0.37 (0.13)	0.46 (0.13)	0.08 (0.12)	28.23 (40.78)	0.11	−1.50
Neutrophils	F 1.91–7.34 M 1.78–6.23	3.11 (0.67)	2.99 (0.87)	−0.12 (1.03)	0.09 (35.83)	2.64 (0.68)	2.89 (0.76)	0.25 (1.05)	15.87 (41.93)	0.83	−0.37
Platelets	F 176–391 M 146–328	227.69 (43.12)	266.00 (52.89)	38.31 (34.47)	17.57 (15.76)	235.75 (52.70)	261.00 (49.83)	25.25 (19.64)	11.55 (8.64)	0.36	0.44

Data are presented as mean (SD) unless otherwise indicated. ^*^No generally accepted standard reference value available. ^a^*P-*value for difference in change from BL to day 7 for thyme-ivy syrup vs control. Calculated by linear mixed repeated measures model for all parameters except IFN-γ, which was calculated by Wilcoxon rank sum test. ^b^Values were not normally distributed. ^c^Cohen’s d for treatment differences in change from BL (negative values indicate the size of the change is larger in the control group). For non-normally distributed data (IFN-γ), the median difference was used as effect size. BL, baseline, CRP, C-reactive protein, F, female; IFN, interferon; IL, interleukin; M, male; SD, standard deviation; TNF, tumor necrosis factor.

Both the thyme-ivy syrup and control groups showed reductions in levels of immune mediators at day 7. Blood cell numbers showed mostly minor changes (increases or decreases) during this time period, but tended to decrease in the thyme-ivy syrup group (except for lymphocytes and platelets) and increase in the control group.

In exploratory statistical analyses, changes from BL to day 7 in immune mediators and blood cells were not statistically different between the thyme-ivy syrup and control group (Table 2), although the decrease in IL-6 showed the largest treatment difference (mean −4.9 [SD 5.4] for thyme-ivy syrup vs. − 0.9 [SD 1.9] in the control group; *p* = 0.09 for treatment effect in LMM). The decrease in IL-10 was also numerically greater in the thyme-ivy group (mean −17.67 [SD 27.14] vs. − 5.95 [SD 6.61]). Treatment by visit interaction was only significant for eosinophils *p* = 0.0159. All other immune response mediators also showed greater reductions in the thyme-ivy syrup vs. control group, although differences did not reach statistical significance (Table 2).

Notably, comparing the change from BL to day 7 in IL-6 and TNF compared between the treatment groups, a large effect size (Cohen’s d) was observed (0.87 and 0.88, respectively) in favor of the thyme-ivy syrup group. Other immune response parameters, such as CRP, IFN-γ, IL-8, and IL-10, showed a moderate effect size (d ≥ 0.5) (Table 2) in favor of the thyme-ivy syrup group.

### 3.3 Secondary efficacy endpoints: additional blood parameters

Secondary efficacy evaluations included various blood parameters at all study visits. Mean values (Supplementary Table S3) and changes from BL were generally similar between the thyme-ivy syrup and control groups. However, four significant differences between groups in change from BL were identified, three at day 4 (IL-10, hemoglobin, and prothrombin time) and one at day 14 (eosinophil counts) (Table 3). For IL-10, the thyme-ivy syrup group showed a greater decrease than the control group, and for eosinophil counts, the thyme-ivy syrup group decreased compared with an increase in the control group. Hemoglobin levels at day 4 decreased in the control group and increased slightly in the thyme-ivy syrup group, while prothrombin time increased more in the thyme-ivy syrup group (Table 3).

**Table 3:** Significant differences for change from BL observed for blood parameters in secondary efficacy analyses.

Parameter and group	BL value	Day	Value on specified study day	Absolute change from BL to specified study day	% change from BL to specified day	*P*-value^a^
IL-10, pg/mL						
Thyme-ivy syrup	25.02 (27.31)	4	10.26 (3.16)	−14.75 (26.28)	−44.08 (20.12)	0.028
Control	11.16 (6.39)	4	5.82 (2.55)	−5.35 (6.01)	−29.62 (60.44)	
Eosinophils, per nL						
Thyme-ivy syrup	0.18 (0.13)	14	0.13 (0.08)	−0.043 (0.115)	24.53 (148.25)	0.009
Control	0.15 (0.17)	14	0.20 (0.14)	0.045 (0.059)	108.71 (149.08)	
Hemoglobin, mmol/L						
Thyme-ivy syrup	8.89 (0.72)	4	8.90 (0.61)	0.008 (0.32)	0.23 (3.42)	0.031
Control	8.63 (0.68)	4	8.40 (0.62)	−0.23 (0.20)	−2.55 (2.21)	
Prothrombin time, %						
Thyme-ivy syrup^b^	90.67 (7.97)	4	95.27 (8.36)	6.0 (8.06)	7.01 (0.56)	0.024
Control	97.13 (12.69)	4	96.63 (9.04)	−0.50 (7.25)	0.03 (6.65)	

Data are presented as mean (SD). ^a^Calculated by linear mixed repeated measures model for IL-10 and eosinophils and by Wilcoxon rank sum test for hemoglobin and prothrombin time. ^b^One missing value at both timepoints. BL, baseline; IL, interleukin; SD, standard deviation.

### 3.4 Secondary efficacy endpoints: changes in symptoms and SARS-CoV-2-negative status

**Figure 2:**
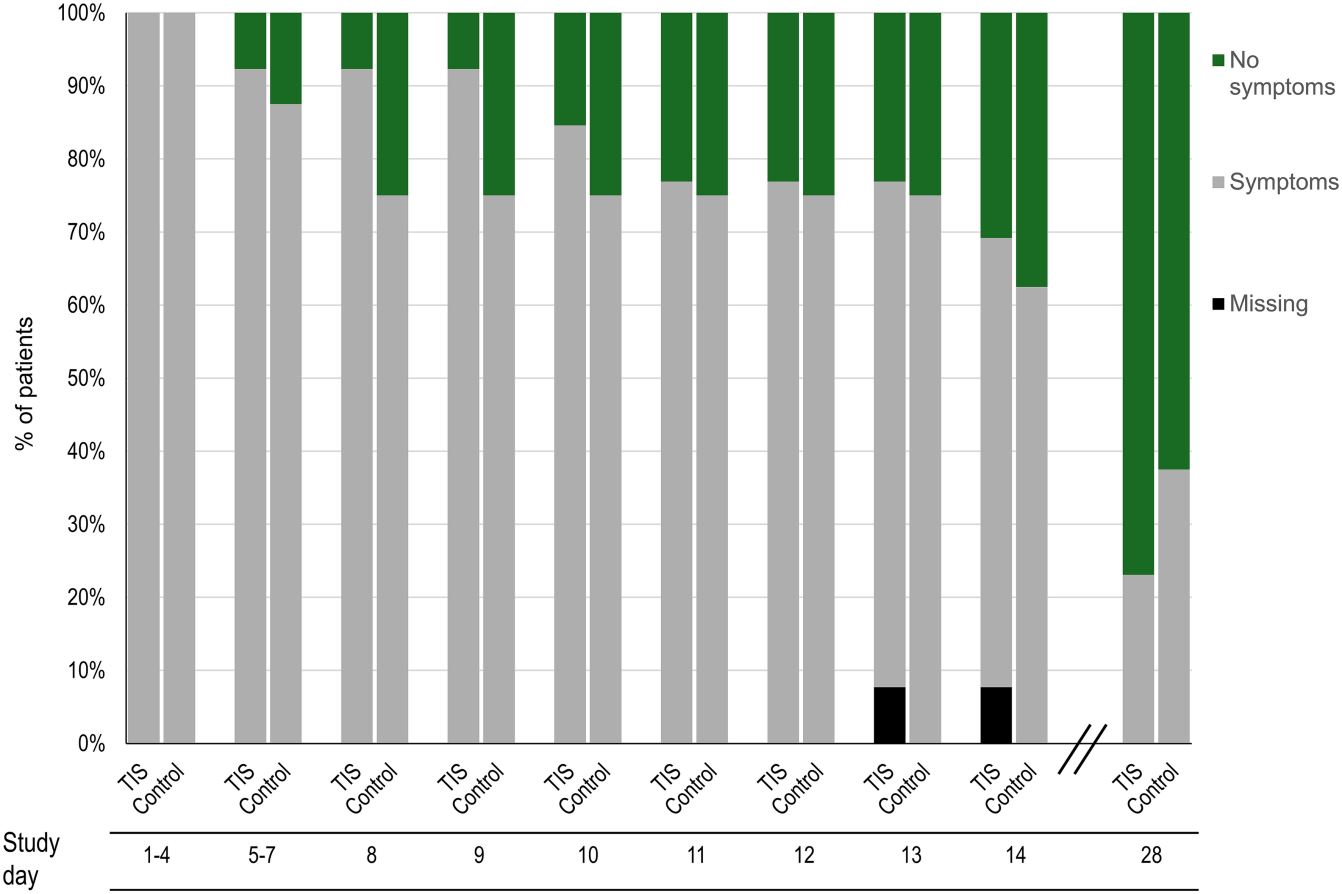
Percentages of patients reporting “no symptoms” based on the FDA 14-symptom questionnaire during the study time period in the thyme-ivy syrup (*n =* 13) and control (*n =* 8) groups. FDA, US Food and Drug Administration; TIS, thyme-ivy syrup.

At BL, the thyme-ivy syrup group had a slightly higher mean (SD) number of symptoms compared with the control group (6.8 [2.5] vs. 5.9 [1.1]). Both the thyme-ivy syrup and control groups showed improvements in symptoms over the 2-week study period (Supplementary Figure S1A). There were no significant differences in the number of symptoms or change in the number of symptoms between groups at BL, day 7, or day 14. The mean severity of symptoms also decreased over a similar time frame in both groups (Supplementary Figure S1B). The time for patients to reach a “no symptom” status was similar between groups (mean [SD] of 21.8 (2.6) for thyme-ivy syrup vs. 21.5 (3.8) for control) (Figure 2). At days 14 and 28, the thyme-ivy syrup group reported a lower mean number of symptoms and lower mean symptom severity compared with the control group (Supplementary Figure S1). At the 28-day follow-up visit, 10/13 (76.9%) patients in the thyme-ivy syrup group and 5/8 (62.5%) patients in the control group reported no symptoms.

Analyses of specific symptoms found that the types and numbers of symptoms at BL and day 7 were generally similar between the two groups (Supplementary Table S4). A larger proportion of patients in the thyme-ivy syrup group reported moderate/severe cough at BL (10/13 [76.9%] vs. 4/8 [50.0%] in the control group) (Supplementary Table S5). Overall resolution of cough was similar: 12/13 (92.3%) of patients in the thyme-ivy syrup group and 8/8 (100%) of patients in the control group reported no cough at the 28-day follow-up visit, and the mean (SD) time from symptom start to no cough was also comparable (18.7 [2.8] vs. 20.8 [4.7] days). However, the mean (SD) time from symptom start to a mild or non-existent cough was longer in the thyme-ivy syrup group (9.1 [1.1] days vs. 6.5 [0.5] for the control group).

With respect to the most bothersome symptom, at BL cough was more frequently reported in the thyme-ivy syrup group compared with control (6 patients vs. 2 for cough as the single most bothersome symptom; 8 vs. 3 for cough alone or combined with other symptoms) (Table 1). The mean intensity of the most bothersome symptom was similar between groups at BL (47.0 [22.9] for thyme-ivy syrup and 47.5 [23.4] for control on a 100-mm VAS) and decreased similarly in both groups throughout the study.

The quality-of-life questions concerning return to usual health and return to usual activity indicated that recovery patterns were generally similar in the two groups (Supplementary Figure S2). Although the control group showed a slightly faster trajectory for return to usual health, the thyme-ivy syrup group had a similar percentage by day 11 and higher proportions of patients at subsequent time points compared with control, including day 28 (84.6% vs. 75.0% for control reported a return to usual health) (Supplementary Figure S2). Changes in WHO score during the study period were also generally comparable in the two groups, although again achieved somewhat more quickly in the control group. At the screening examination and at day 4, all patients reported a WHO score of 2. Improvements to scores <2 were observed in 3/13 (23.1%) patients in the thyme-ivy syrup and 3/8 (37.5%) in the control group at day 7, 7/13 (53.8%) and 7/8 (87.5%) at day 14, and 12/13 (92.3%) and 7/8 (87.5%) at day 28. These patterns were supported by an analysis of SARS-CoV-2 negative status, which indicated a longer duration of SARS-CoV-2 infection in the thyme-ivy syrup group compared with the control group (mean [standard error] of 11.5 (1.3) vs. 8.0 (1.5) days from study initiation; *p* = 0.032).

### 3.5 Secondary efficacy endpoints: concomitant medication due to COVID-19 symptoms

In the thyme-ivy syrup group, 3 patients received concomitant medication due to COVID-19 symptoms: 1 patient received both xylometazoline and paracetamol, 1 received xylometazoline only, and 1 received paracetamol only. For xylometazoline nasal spray, 1 patient was receiving treatment with this drug at BL for COVID-19 symptoms and an additional patient began taking xylometazoline during the study. The mean (SD) days of xylometazoline intake for these 2 patients was 7.5 (0.5). The 2 patients who received paracetamol for COVID-19 symptoms during the study period reported a maximum dose of 3,000 mg for a mean (SD) of 0.3 (0.9) days. No patients in the control group received concomitant medications for COVID-19 symptoms.

### 3.6 Safety

No patients in either group required hospitalization, oxygen supplementation, or withdrawal from the study/study medication during the study period. In the thyme-ivy syrup group, 6 AEs were reported in 4 patients; 2 of these patients each reported 2 AEs (urticaria and tachycardia in one and dyspnea and headache in the other), and the other 2 patients each reported 1 AE. In the control group, 2 patients each reported 1 AE for a total of 2 AEs (Table 4). All AEs were mild except for one moderate case of urticaria in a patient receiving thyme-ivy syrup. Most of the symptoms (nasopharyngitis, headache, dyspnea) were likely related to the SARS-CoV-2 infection. Only one AE, anemia in the thyme-ivy syrup group, was considered by the investigator to be related to treatment. No further information could be obtained from the treating physician to sustain this assessment. All AEs resolved without sequelae during the study period.

**Table 4:** Adverse event reports over 28 days.

Adverse event by SOC/PT	Thyme-ivy syrup (*n =* 13)	Control (*n =* 8)
Blood and lymphatic system disorders		
Anemia	1 (7.7%)	0
Cardiac disorders		
Tachycardia	1 (7.7%)	0
Infections and infestations		
Nasopharyngitis	1 (7.7%)	1 (12.5%)
Nervous system disorders		
Headache	1 (7.7%)	0
Respiratory, thoracic and mediastinal disorders		
Dyspnea	1 (7.7%)	1 (12.5%)
Skin and subcutaneous tissue disorders		
Urticaria	1 (7.7%)	

Displayed are the number (*n*) and percentage (%) of patients experiencing AEs. In the thyme-ivy syrup group, 6 AEs occurred in 4 patients. Two patients each reported 2 AEs; one patient reported urticaria and tachycardia and the other patient reported dyspnea and headache. All other patients reported single AEs. In the control group, 2 AEs occurred in 2 patients. PT, preferred term; SOC, system organ class.

## 4 Discussion

This randomized, open-label, pilot study of thyme-ivy syrup in adults with mild COVID-19 was designed to provide information for a subsequent larger confirmatory clinical trial. Accordingly, this study was not powered to identify statistical significances, but to provide insights into immune response mediators that might be affected to a greater degree by thyme-ivy syrup compared to not receiving thyme-ivy syrup.

Although a phase IV clinical trial demonstrated the efficacy of thyme-ivy syrup in reducing symptoms associated with acute bronchitis, particularly cough (9), to the best of our knowledge this is the first study to evaluate the effect of thyme-ivy syrup on immune response mediators and blood cell types in humans as well as the first to study this drug in patients with confirmed SARS-CoV-2 infection. An assessment of immune response mediators is particularly relevant in patients with SARS-CoV-2 as dysregulation of the immune system is a key characteristic of COVID-19 (31).

Overall BL demographics were generally well-matched between the thyme-ivy syrup and control group, but there were differences in BL symptoms and markers of infection. Compared with mean values in the control group, the thyme-ivy syrup group had a shorter time from symptom onset to the BL visit, higher viral loads, higher numbers of symptoms, greater symptom severity, and higher levels of certain immune response mediators at BL. It is possible that these differences impacted day 7 outcomes and comparisons with the control group.

In this pilot study, we identified treatment differences in change from BL to day 7 in immune response mediators for the thyme-ivy syrup vs. control group. Numerically greater decreases were observed for several key molecules, particularly CRP, IL-6, IL-8, IL-10, and TNF. These differences were not statistically significant, but the study was not powered to identify statistically significant differences. However, BL values for these immune response mediators were higher in the thyme-ivy syrup group compared with the control group, which allowed the potential for greater decreases from BL. At day 4, levels of IL-10 showed a significant decrease from BL in the thyme-ivy syrup group compared with the control group, but this may also have been influenced by BL levels. IL-10 is a multifunctional cytokine that acts as both a pro- and anti-inflammatory cytokine (31). Higher IL-10 levels have been associated with more severe COVID-19 (32, 33).

A moderate to large effect size was observed in comparisons of changes from BL to day 7 between the study groups in favor of thyme-ivy syrup. For IL-6 and TNF, a substantial effect size was observed (0.87 and 0.88, respectively). In contrast, for other immune response parameters, such as CRP, IFN-γ, IL-8, and IL-10, a moderate effect size was observed (d ≥ 0.5) in favor of thyme-ivy syrup. Due to the observed differences between the groups at BL and the non-significant treatment differences, it is likely that the effect sizes have been inflated. However, if a *p*-value in this pilot study is below 0.1 (10% significance level, e.g., for IL-6) and the effect size is moderate, the impact of the treatment is likely, and the lack of significance may be related to the small sample size. Accordingly, in consideration of the findings, the most promising cytokine that may exhibit treatment-related variations is IL-6, with a *p*-value of 0.09 and an effect size of 0.87. Therefore, this cytokine may serve as a promising marker for additional confirmatory studies.

In the literature, clinically relevant changes or differences are mainly defined by statistical significance. However, these significant changes or differences can vary greatly among cytokines and among studies based on indication, patient population, and assays (34). Chen et al. (35) reported significantly different IL-6 levels between mild and severe COVID-19 (34 ± 7 pg/mL vs. 52 ± 11 pg/mL). Along the same lines, Qin et al. (5) reported significantly different IL-6 levels between severe and non-severe COVID-19 cases (25.2 pg/mL vs. 13.3 pg/mL, *p* < 0.001) as well as significantly different IL-10 levels between these cases (6.6 pg/mL vs. 5.0 pg/mL; *p* < 0.001). Further, Wu et al. (36) reported significant differences for patients progressing to ARDS versus those not progressing to ARDS for IL-6 (7.39 pg/mL vs. 6.29 pg/mL, *p* = 0.03). These examples suggest that a significant difference of 1.1 pg/mL for IL-6 or 1.6 pg/mL for IL-10 can be observed in comparable settings and may indicate clinical relevance.

With respect to changes in blood cells during thyme-ivy syrup treatment, pre-clinical studies in a rat model of bronchitis found that thyme-ivy syrup was associated with significant decreases in leukocyte and granulocyte counts in bronchial fluid and peripheral blood after lipopolysaccharide stimulation compared with controls (37). In our study, we observed decreases in specific types of granulocytes (basophils, eosinophils, and neutrophils) in patients receiving thyme-ivy syrup at day 7, whereas blood counts for these cells increased slightly in the control group. There were no significant between-group differences in change from BL to day 7 in blood cell counts, but for eosinophils the change from BL to day 14 was significantly different between the groups (decreased counts in the thyme-ivy syrup group vs. increased counts in the control group).

It is unclear whether the significant between-group differences in change from BL in hemoglobin levels and prothrombin time observed at day 4 have clinical relevance, as changes were relatively minor. However, SARS-CoV-2 infection is known to alter biomarkers of iron metabolism (38) and thrombosis (39). It is therefore possible that these data reflect true physiological effects associated with thyme-ivy syrup.

Changes in symptoms were similar between the thyme-ivy syrup and control groups; in both groups, symptoms resolved fairly rapidly as is typical for cases of mild COVID-19 (40, 41). At the 28-day follow-up, the majority of patients reported no symptoms. Return to usual health and usual activity occurred more quickly in the control group, probably due to the lower disease severity in these patients and the longer time between symptom onset and the BL visit. However, by day 14 these differences had largely disappeared.

A larger proportion of patients in the thyme-ivy syrup group reported moderate to severe cough at BL and considered cough to be the most bothersome symptom. Despite the efficacy of thyme-ivy syrup in reducing cough and other symptoms associated with acute bronchitis (9), differences between groups in cough resolution were minimal. Between-group differences may have been masked by the greater proportion of patients with cough in the thyme-ivy syrup group at BL. However, there are multiple pathways by which respiratory viruses can promote cough (42), and there remains much to be learned about the mechanisms by which SARS-CoV-2 causes cough (43). It is possible that cough associated with SARS-CoV-2 infections employ different pathophysiological pathways than those used by other common respiratory viruses. For instance, a major pathway by which thyme-ivy syrup improves cough symptoms is through mucus-regulatory activity, including effects on goblet cells (10, 37), and this pathway may be of lesser importance in SARS-CoV-2-associated cough.

Thyme-ivy syrup was well tolerated during this study and there were no safety signals of concern. Although one adverse event, anemia, was considered related to treatment, no further information could be obtained from the treating physician to sustain this assessment. Moreover, there were no reports of anemia in large-scale clinical trials of thyme-ivy syrup in acute bronchitis (9), whereas anemia is fairly common in patients with COVID-19 (44). Blood analyses of potential markers of thrombosis showed no clinically relevant differences between the thyme-ivy syrup and control groups.

Limitations of our pilot study include a small sample size due to the termination of routine SARS-CoV-2 testing at our center. All patients in this study had been vaccinated against COVID-19, which likely influenced blood parameters involved in the immune response. Due to the exploratory nature of this study, there were no adjustments for disease severity; as discussed above, BL data suggest that the thyme-ivy syrup group had more severe disease and a heavier symptom burden at study start compared with the control group. The study was not blinded, but the open-label nature of the study did not appear to influence the results. The patient population in this study was limited to patients with mild COVID-19. We hope that researchers are able to build on these results when designing studies of phytotherapeutic use in a wider range of populations, including asymptomatic COVID-19 patients and those affected by more serious COVID-19 or other diseases.

The small sample size and the potentially heavier burden of disease in the treatment group may have obscured changes in the outcomes evaluated, particularly in the context of the wide heterogeneity observed in inflammatory markers in patients with COVID-19 (45, 46). It is also possible that the 7-day timepoint chosen for the primary efficacy endpoint was too late to detect treatment-related changes over the background of improvements mediated by the immune system, especially given that all patients in the study had been vaccinated against SARS-CoV-2 infection.

Differences in BL symptoms and markers of infection in the thyme-ivy syrup group, such as shorter time from symptom onset to the BL visit, higher viral loads, higher numbers of symptoms, greater symptom severity, and higher levels of certain immune response mediators at BL might have impacted day 7 outcomes and comparisons with the control group.

## 5 Conclusion

The results of this pilot study identified treatment changes in some immune response mediators in the thyme-ivy syrup group vs. the control group that will need to be validated in a larger confirmatory trial. Although differences were not statistically significant, this is likely due to study design, as this study was not designed or powered for detecting statistical significance. Nevertheless, some of the changes we observed suggest clinically relevant changes in the thyme-ivy group. Baseline effects need to be taken into account when modelling a larger trial, as we have seen their potential impact on the interpretation of treatment effects.

Our pilot study provides reassuring insights into the safety of thyme-ivy syrup in this patient population. Although future studies with larger and more balanced patient populations will be required for definitive conclusions about the effects of thyme-ivy syrup on blood parameters and symptoms, our study supports the overall safety of thyme-ivy syrup in patients with mild COVID-19 and offers preliminary insights into its effects on immune response mediators and blood cells.

## Data Availability

The raw data supporting the conclusions of this article will be made available by the authors, without undue reservation.
