# Supplementary material for: Effect of thyme-ivy syrup on antiviral immune response in patients with mild COVID-19: a prospective, open-label, randomized pilot study
Source: Front Med (Lausanne). 2025 Oct 22;12:1672794. doi: 10.3389/fmed.2025.1672794 (PMC12585981; doi:10.3389/fmed.2025.1672794)

Supplementary Material

**TABLE S1** Concomitant medications that were not allowed up to study day 14.

| **Unpermitted medications** |
| --- |
| - Any COVID-19 medication (monoclonal antibodies, remdesivir, antiviral substances) |
| - Acetylsalicylic acid (>300 mg/day) |
| - Aspecton |
| - Bronchicum |
| - Bronchostop |
| - Coxibs: celecoxib, etoricoxib, parecoxib |
| - Dexamethasone |
| - Diclofenac, |
| - Gelomyrthol |
| - Hydrocortisone |
| - Ibuprofen |
| - Mucosolvan, |
| - Naproxen, |
| - Prednisone/prednisolone |
| - Prospan |
| - Remdesvir |
| - Silomat |
| - Umckaloabo |

**TABLE S2** Entry criteria for the BroVID study.

| **Inclusion criteria** |
| --- |
| - ≥18 and ≤75 years of age   - If >50 years, complete COVID-19 vaccination mandatory |
| - SARS-CoV-2 infection confirmed by PCR test ≤4 days before screening/baseline visit |
| - Onset of the earliest symptoms <7 days before screening/baseline visit |
| - Mild COVID-19 with the following symptoms (outpatient management/non-hospitalized patients)   - Cough and   - At least one other symptom (eg, sore throat, nasal congestion, headache nausea, low energy/fatigue, muscle or body ache, shortness of breath, fever, diarrhea, altered sense of smell or taste) |
| - Written informed consent obtained prior to the initiation of any protocol-required procedures by the patient |
| - Willingness to comply with study procedures and protocol |
| **Exclusion criteria** |
| - WHO score ≥3 |
| - Other advanced or chronic lung disease (chronic obstructive pulmonary disease, silicosis, bronchial asthma) |
| - BMI >35 kg/m^2^ or body weight <43 kg |
| - Requirement for oxygen administration |
| - Current hospitalization |
| - Known hypersensitivity to the active substances, including ivy, thyme, plants of the aralia family or other labiates (Lamiaceae), birch, mugwort, celery or to any of the excipients |
| - Rare hereditary fructose intolerance |
| - Inability to monitor body temperature |
| - Currently taking immunosuppressive medication, non-steroidal anti-inflammatory drugs, or steroids (eg, because of an underlying disease) |
| - Known significant concomitant diseases or serious and/or uncontrolled diseases that are likely to interfere with the evaluation of the patient’s safety and with the study outcome, such as stem cell or organ transplantation within the last 5 years, cardiovascular disease, diabetes mellitus, chronic liver disease chronic kidney disease including dialysis patient, sickle cell anemia or thalassemia, and other forms of immunosuppression (eg., tumor patients, human immunodeficiency virus-infected patients with weakened immune systems, iatrogenic immunosuppression) as judged by the study physician according to the patient’s reports |
| - COVID-19 vaccination planned within the study period and/or within the last 28 days |
| - Women pregnant (patient-reported at pre-screening and confirmation via pregnancy test at screening/baseline visit) or nursing |
| - Males or females of reproductive potential not willing to use effective contraception (defined as PEARL index <1, eg, contraceptive pill, IUD) |
| - Alcohol, drug, or chemical abuse |
| - Current participation in another interventional clinical trial |

Supplementary Material should be uploaded separately on submission. Please include any supplementary data, figures and/or tables.

**TABLE S3** Blood parameters investigated as secondary endpoints in the thyme-ivy syrup (n=13) and control (n=8) groups, including blood parameters evaluated as primary endpoints at additional time points. Statistically significant differences between groups in change from BL are shaded in blue and shown in Table 3. Data are presented as mean (SD) unless otherwise indicated.

| **Parameter** | **Normal range** | **BL (day 0)** | | **Day 4** | | **Day 7** | | **Day 14** | |
| --- | --- | --- | --- | --- | --- | --- | --- | --- | --- |
|  |  | **Thyme-ivy syrup** | **Control** | **Thyme-ivy syrup** | **Control** | **Thyme-ivy syrup** | **Control** | **Thyme-ivy syrup** | **Control** |
| CRP, mg/L | F/M < 5.0 | 5.49 (4.27) | 3.29 (2.25) | 2.36 (1.84) | 1.08 (0.45) | 1.25 (1.24) | 0.90 (0.81) | 0.90 (1.02) | 0.90 (0.99) |
| IFN-γ, median (min, max) pg/mL^a^ | * | 0.98  (0.09, 4.40) | 0.45  (0.14, 1.31) | 0.17  (0.08, 0.43) | 0.20  (0.09, 0.35) | 0.14  (0.08, 0.35) | 0.21  (0.11, 0.28) | 0.17  (0.10, 0.32) | 0.15  (0.09, 0.37) |
| IL-6, pg/mL | * | 6.64 (5.80) | 3.04 (1.93) | 2.04 (1.38) | 4.08 (6.59) | 1.78 (1.02) | 2.09 (1.07) | 2.03 (1.71) | 1.75 (0.62) |
| IL-8, pg/mL | * | 6.11 (3.18) | 4.72 (1.41) | 4.85 (1.20) | 4.41 (1.29) | 4.22 (1.15) | 4.14 (1.10) | 4.33 (1.29) | 4.15 (0.83) |
| IL-10, pg/mL | * | 25.02 (27.31) | 11.16 (6.39) | 10.26 (3.16) | 5.82 (2.55) | 7.35 (2.66) | 5.21 (2.57) | 7.01 (2.18) | 5.43 (1.40) |
| TNF, pg/mL | * | 23.23 (6.95) | 16.51 (2.78) | 18.71 (4.95) | 16.04 (2.36) | 16.32 (4.65) | 14.19 (2.11) | 16.36 (3.45) | 13.61 (3.04) |
| Ferritin, µg/L | F 15.0 – 150.0  M 30.0 – 400.0 | 120.27 (74.99) | 105.99 (46.37) | 122.13 (85.03) | 116.53 (42.15) | 118.05 (19.20) | 102.73 (44.65) | 103.36 (68.80) | 87.19 (36.12) |
| Hemoglobin, mmol/L | F 7.2 – 9.7  M 8.4 – 10.9 | 8.89 (0.72) | 8.63 (0.68) | 8.90 (0.61) | 8.40 (0.62) | 8.65 (0.72) | 8.30 (0.62) | 8.57 (0.76) | 8.06 (0.84) |
| IgG, g/L | F/M 7.0 – 16.0 | 11.55 (1.47) | 13.15 (1.91) | 11.52 (1.45) | 12.95 (1.95) | 11.51 (1.42) | 12.95 (1.98) | 11.51 (1.34) | 12.48 (2.13) |
| IgM, g/L | F/M 0.40 – 2.30 | 1.07 (0.42) | 1.45 (0.58) | 1.07 (0.41) | 1.40 (0.55) | 1.04 (0.39) | 1.40 (0.53) | 1.02 (0.38) | 1.32 (0.54) |
| AST, U/L | F < 35  M < 50 | 21.08 (3.99) | 21.75 (4.71) | 20.31 (4.31) | 21.25 (3.95) | 23.77 (6.06) | 20.38 (3.74) | 20.85 (3.80) | 20.13 (4.97) |
| ALT, U/L | F < 35  M < 50 | 21.77 (5.97) | 25.13 (14.09) | 20.31 (5.07) | 26.25 (16.21) | 23.00 (7.35) | 22.50 (10.92) | 20.85 (4.81) | 19.13 (6.75) |
| Creatinine, µmol/L | F 44.2-79.6  M 61.9-106.1 | 79.49 (16.65) | 73.36 (12.93) | 79.55 (16.93) | 74.81 (11.30) | 79.15 (14.30) | 78.79 (12.50) | 80.50 (18.99) | 78.68 (11.23) |
| aPTT, sec | F/M 26.0-40.0 | 36.68 (3.03)^b^ | 36.78 (2.05) | 35.64 (2.32)^b^ | 37.05 (2.67) | 36.24 (2.76) | 36.88 (2.93) | 35.72 (2.51) | 37.33 (2.61) |
| Prothrombin time, % | F/M >70 | 90.67 (7.97)^b^ | 97.13 (12.69) | 95.27 (8.36)^b^ | 96.63 (9.04) | 93.00 (9.85) | 92.63 (10.17) | 90.85 (8.27) | 90.63 (10.45) |
| Fibrinogen, g/L | F/M 2.00-4.50 | 3.45 (0.56) | 3.74 (0.57) | 3.43 (0.46) | 3.31 (0.49) | 2.95 (0.46) | 3.17 (0.50) | 2.67 (0.47) | 2.94 (0.48) |
| Anti-thrombin III activity, % | F/M 83-128 | 111.07 (8.06) | 107.75 (9.48) | 112.45 (8.23) | 106.88 (6.85) | 109.46 (9.40) | 103.75 (9.48) | 110.54 (11.54) | 102.38 (11.39) |
| Basophils per nL | F/M < 0.08 | 0.04 (0.02) | 0.03 (0.01) | 0.03 (0.01) | 0.03 (0.01) | 0.04 (0.01) | 0.04 (0.01) | 0.05 (0.02) | 0.04 (0.01) |
| Eosinophils per nL | F/M 0.03 – 0.44 | 0.18 (0.13) | 0.15 (0.17) | 0.17 (0.11) | 0.15 (0.18) | 0.15 (0.10) | 0.16 (0.10) | 0.13 (0.08) | 0.20 (0.14) |
| Lymphocytes per nL | F 1.22 – 3.56  M 1.05 – 3.24 | 1.49 (0.36) | 1.66 (0.24) | 1.76 (0.49) | 1.96 (0.46) | 1.88 (0.56) | 2.16 (0.61) | 1.89 (0.63) | 1.85 (0.29) |
| Monocytes per nL | F 0.25 – 0.85  M 0.26 – 0.87 | 0.61 (0.14) | 0.37 (0.13) | 0.39 (0.11) | 0.37 (0.14) | 0.44 (0.10) | 0.46 (0.13) | 0.49 (0.12) | 0.44 (0.17) |
| Neutrophils per nL | F 1.91 – 7.34  M 1.78 – 6.23 | 3.11 (0.67) | 2.64 (0.68) | 2.60 (0.84) | 2.72 (0.84) | 2.99 (0.87) | 2.89 (0.76) | 3.36 (1.50) | 2.59 (0.71) |
| Platelets per nL | F 176-391  M 146 - 328 | 227.69 (43.12) | 235.75 (52.70) | 241.08 (49.52) | 242.00 (50.53) | 266.00 (52.89) | 261.00 (49.83) | 272.00 (53.31) | 244.13 (49.96) |

^a^Values were not normally distributed. Median, min, and max are presented for this parameter.

^b^One missing value.

* No generally accepted standard reference value available.

ALT, alanine aminotransferase; aPTT, activated partial thromboplastin time; AST, aspartate aminotransferase; BL, baseline; CRP, C-reactive protein; F, female; IFN, interferon; Ig, immunoglobulin; IL, interleukin; LDH, lactate dehydrogenase; M, male; SD, standard deviation; TNF, tumor necrosis factor

**TABLE S4** Numbers of patients experiencing specific symptoms (based on the FDA symptom questionnaire) at baseline and day 7.

| **Symptom** | **Thyme-ivy syrup (n=13)** | | **Control (n=8)** | |
| --- | --- | --- | --- | --- |
|  | **Baseline** | **Day 7** | **Baseline** | **Day7** |
| Stuffy or runny nose | 13 | 11 | 8 | 6 |
| Sore throat | 9 | 4 | 5 | 0 |
| Shortness of breath | 5 | 3 | 4 | 2 |
| Cough | 13 | 10 | 8 | 4 |
| Low energy and tiredness | 12 | 10 | 7 | 4 |
| Muscle or body aches | 9 | 1 | 2 | 1 |
| Headache | 10 | 2 | 5 | 2 |
| Chills or shivering | 2 | 0 | 1 | 0 |
| Feeling hot or feverish | 5 | 0 | 0 | 0 |
| Nausea | 2 | 0 | 0 | 0 |
| Vomiting within last 24 hours | 0 | 0 | 0 | 0 |
| Diarrhea within last 24 hours | 2 | 1 | 0 | 0 |

**TABLE S5** Patient reports of cough and cough severity during the study period. Data are presented as numbers of patients.

| **Study day** | **Thyme-ivy syrup**  **(n=13)** | | | | **Control**  **(n=8)** | | | |
| --- | --- | --- | --- | --- | --- | --- | --- | --- |
|  | **No cough** | **Mild cough** | **Moderate/**  **severe cough** | **Missing** | **No cough** | **Mild cough** | **Moderate/**  **severe cough** | **Missing** |
| 1 | 0 | 3 | 10 | 0 | 0 | 4 | 4 | 0 |
| 2 | 0 | 3 | 10 | 0 | 0 | 7 | 1 | 0 |
| 3 | 1 | 6 | 6 | 0 | 3 | 4 | 1 | 0 |
| 4 | 2 | 5 | 6 | 0 | 3 | 4 | 1 | 0 |
| 5 | 3 | 4 | 6 | 0 | 4 | 4 | 0 | 0 |
| 6 | 3 | 4 | 6 | 0 | 4 | 2 | 2 | 0 |
| 7 | 3 | 5 | 5 | 0 | 4 | 3 | 1 | 0 |
| 8 | 4 | 7 | 2 | 0 | 4 | 3 | 1 | 0 |
| 9 | 4 | 7 | 2 | 0 | 4 | 4 | 0 | 0 |
| 10 | 5 | 6 | 2 | 0 | 4 | 3 | 1 | 0 |
| 11 | 5 | 6 | 2 | 0 | 4 | 3 | 1 | 0 |
| 12 | 6 | 6 | 1 | 0 | 4 | 3 | 1 | 0 |
| 13 | 5 | 7 | 0 | 1 | 4 | 3 | 1 | 0 |
| 14 | 8 | 4 | 0 | 1 | 4 | 4 | 0 | 0 |
| 28 | 12 | 1 | 0 | 0 | 8 | 0 | 0 | 0 |

**Supplementary Figure S1.** Boxplots of changes in (A) number of symptoms (based on 14 FDA questionnaire symptoms) and (B) mean total symptom severity per symptom (based on 10 FDA questionnaire symptoms rated from none=0 to severe=3) over time. The top and bottom of the boxes correspond to Q3 and Q1, respectively, the horizontal line in the box indicates the median, and the X indicates the mean. Vertical lines (whiskers) show the maximum and minimum without outliers; outliers are indicated by dots.


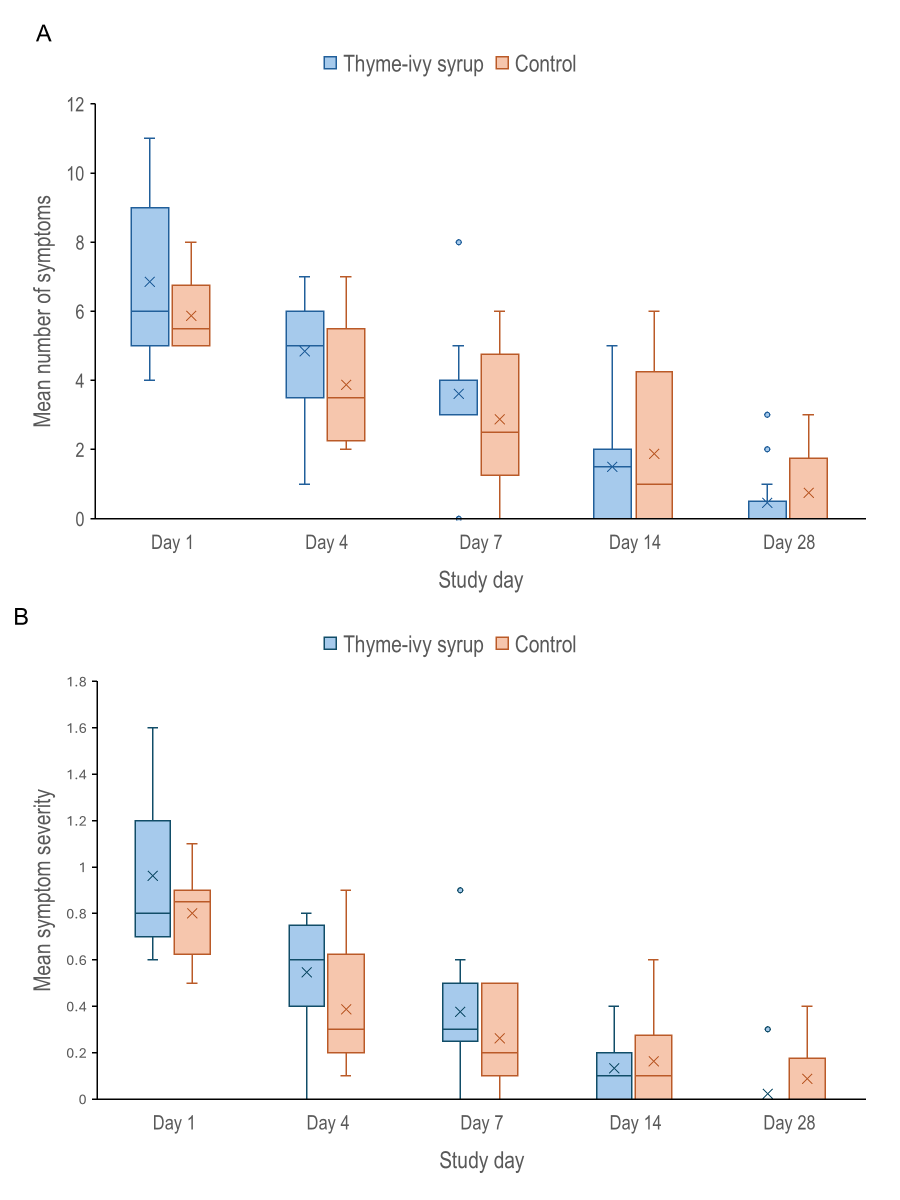


**Supplementary Figure S2** Quality of life assessments: Percentages of patients reporting (A) return to usual health and (B) return to usual activity. Squares indicate 28-day follow-up visit.


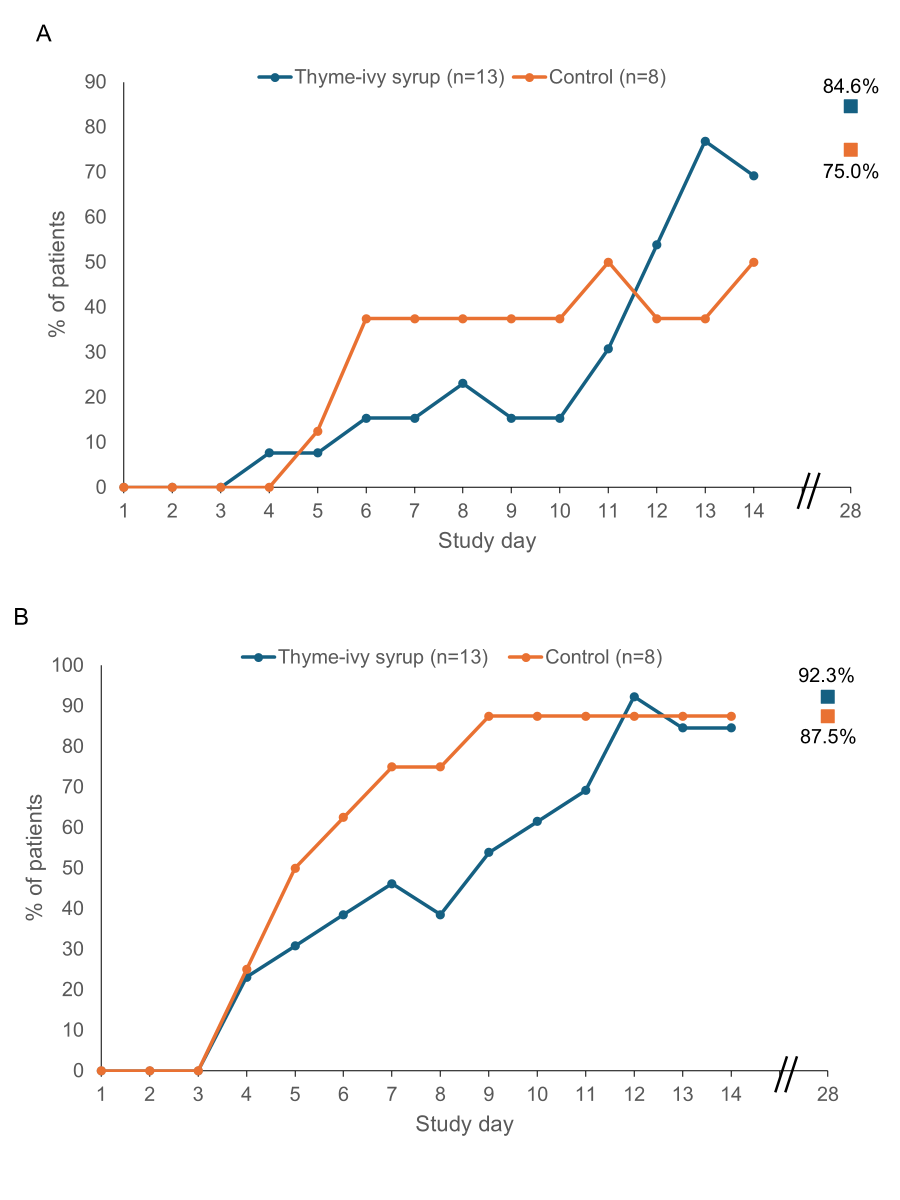

Supplement: Supplementary file 1 [file Data_Sheet_1.docx]
