# Supplementary material for: Effect of thyme-ivy syrup on antiviral immune response in patients with mild COVID-19: a prospective, open-label, randomized pilot study
Source: Front Med (Lausanne). 2025 Oct 22;12:1672794. doi: 10.3389/fmed.2025.1672794 (PMC12585981; doi:10.3389/fmed.2025.1672794)
Supplement: Supplementary file 2 [file Data_Sheet_2.docx]

Supplementary Material

# Supplementary Appendix: Linear mixed model effects for the FAS (imputed)

LMM data for each blood parameter includes the estimated treatment effect size at day 7 (visit 3), treatment comparison estimated as linear contrast at day 7 with p values, and tested effects of the model with p-values.

For the following blood parameters no LMM was constructed, reasons given

| **Blood Parameter w/o LMM** |  | **Comment** |
| --- | --- | --- |
| Human IFNalpha | 98.81 % of data are missing or BLOQ/ALOQ. Refer to summary statistics |  |
| IFNG [pg/ml] | Model fit failed, despite Box-Cos transformation. Refer to Wilcoxon test. | See table below |
| IL1B | 95.24 % of data are missing or BLOQ/ALOQ. Refer to summary statistics |  |
| IL2 | 100.0 % of data are missing or BLOQ/ALOQ. Refer to summary statistics |  |
| IL4 | 100.0 % of data are missing or BLOQ/ALOQ. Refer to summary statistics |  |

|  | | **Wilcoxon Rank Sum test Bronchipret syrup vs. Control group** | | | |
| --- | --- | --- | --- | --- | --- |
| **Analyte** | **Visit** | **Statistic** | **Z** | **Pr < Z** | **Pr > \|Z\|** |
| IFNG[pg/mL] | visit 3 | 100.0000 | 0.8328 | 0.2025 | 0.4049 |

**Basophils[/nl]**

No Box Cox Transformation required

| All covariance structures valid (AR(1), CS, UN), CS chosen, since best following AIC. | | | | | | | | | | **95% Confidence interval (two-sided)** | |  |
| --- | --- | --- | --- | --- | --- | --- | --- | --- | --- | --- | --- | --- |
| **Analyte** | **Visit** | **Test** | **Reference** | **N_Test_** | **N_Reference_** | **LSMT** | **LSMR** | **LSM difference estimate** | **Standard Error** | **Lower** | **Upper** | **p-value** |
| Basophils[/nl] | visit 3 | Bronchipret syrup | Control group | 13 | 8 | 0.000325 | 0.003222 | -0.00290 | 0.004829 | -0.01267 | 0.006873 | 0.5521 |

| **Analyte** | **ContrastsT** | **F value** | **p-value (Pr > F)** |
| --- | --- | --- | --- |
| Basophils[/nl] | Contrast Treatment at visit 3 | 0.36 | 0.5521 |

| **Analyte** | **Effect** | **F value** | **p-value (Pr > F)** |
| --- | --- | --- | --- |
| Basophils[/nl] | visit | 10.65 | 0.0002 |
|  | treatment | 0.01 | 0.9042 |
|  | treatment*visit | 1.33 | 0.2768 |
|  | baseline | 42.12 | <.0001 |

**C Reactive Protein[mg/l]**

Box Cox Transformation required for model fit (Lambda=-0.25, c=0)

All covariance structures valid (AR(1), CS, UN), AR(1) chosen, since best following AIC.

|  | | | | | | | | | | **95% Confidence interval (two-sided)** | |  |
| --- | --- | --- | --- | --- | --- | --- | --- | --- | --- | --- | --- | --- |
| **Analyte** | **Visit** | **Test** | **Reference** | **N_Test_** | **N_Reference_** | **LSMT** | **LSMR** | **LSM difference estimate** | **Standard Error** | **Lower** | **Upper** | **p-value** |
| C Reactive Protein[mg/l] | visit 3 | Bronchipret syrup | Control group | 13 | 8 | 0.3702206 | 0.3077481 | 1.2842242 | 0.3531 | 0.637133 | 3.0058805 | 0.4972 |

| LSMs including confidence limits re-transformed | |  |  |  |
| --- | --- | --- | --- | --- |
| **Analyte** | | **ContrastsT** | **F value** | **p-value (Pr > F)** |
| C Reactive Protein[mg/l] | | Contrast Treatment at visit 3 | 0.47 | 0.4972 |

| **Analyte** | **Effect** | **F value** | **p-value (Pr > F)** |
| --- | --- | --- | --- |
| C Reactive Protein[mg/l] | visit | 10.21 | 0.0003 |
|  | treatment | 0.32 | 0.5810 |
|  | treatment*visit | 0.61 | 0.5494 |
|  | baseline | 6.28 | 0.0218 |

**CXCL8[pg/mL]**

Box Cox Transformation required for model fit (Lambda=-0.75, c=0)

All covariance structures valid (AR(1), CS, UN), AR(1) chosen, since best following AIC.

|  | | | | | | | | | | | | | **95% Confidence interval (two-sided)** | | |  | |
| --- | --- | --- | --- | --- | --- | --- | --- | --- | --- | --- | --- | --- | --- | --- | --- | --- | --- |
| **Analyte** | **Visit** | **Test** | | | **Reference** | **N_Test_** | **N_Reference_** | **LSMT** | **LSMR** | **LSM difference estimate** | **Standard Error** | | **Lower** | | **Upper** | **p-value** | |
| CXCL8[pg/mL] | visit 3 | Bronchipret syrup | | | Control group | 13 | 8 | 0.9313562 | 0.9464144 | 0.983428 | 0.03806 | | 0.9134896 | | 1.0633068 | 0.6609 | |
| LSMs including confidence limits re-transformed | | | |  |  |  |  |  |  |  |  |  |  |  |  |  |  |
| **Analyte** | | | | | **ContrastsT** | | | | | | | | **F value** | | **p-value (Pr > F)** | | |
| CXCL8[pg/mL] | | | | | Contrast Treatment at visit 3 | | | | | | | | 0.20 | | 0.6609 | | |

| **Analyte** | **Effect** | **F value** | **p-value (Pr > F)** |
| --- | --- | --- | --- |
| CXCL8[pg/mL] | visit | 1.52 | 0.2308 |
|  | treatment | 0.03 | 0.8654 |
|  | treatment*visit | 0.39 | 0.6819 |
|  | baseline | 26.14 | <.0001 |

**Eosinophils[/nl]**

Box Cox Transformation required for model fit (Lambda=0, c=0)

All covariance structures valid (AR(1), CS, UN), AR(1) chosen, since best following AIC.

|  | | | | | | | | | | **95% Confidence interval (two-sided)** | |  |
| --- | --- | --- | --- | --- | --- | --- | --- | --- | --- | --- | --- | --- |
| **Analyte** | **Visit** | **Test** | **Reference** | **N_Test_** | **N_Reference_** | **LSMT** | **LSMR** | **LSM difference estimate** | **Standard Error** | **Lower** | **Upper** | **p-value** |
| Eosinophils[/nl] | visit 3 | Bronchipret syrup | Control group | 13 | 8 | 0.9923933 | 1.2332237 | 0.8047147 | 0.1943 | 0.5431282 | 1.192289 | 0.2704 |

| LSMs including confidence limits re-transformed | |  |  |  |
| --- | --- | --- | --- | --- |
| **Analyte** | | **ContrastsT** | **F value** | **p-value (Pr > F)** |
| Eosinophils[/nl] | | Contrast Treatment at visit 3 | 1.25 | 0.2704 |

| **Analyte** | **Effect** | **F value** | **p-value (Pr > F)** |
| --- | --- | --- | --- |
| Eosinophils[/nl] | visit | 1.08 | 0.3490 |
|  | treatment | 1.57 | 0.2251 |
|  | treatment*visit | 4.62 | 0.0159 |
|  | baseline | 30.09 | <.0001 |

**IL10**

Box Cox Transformation required for model fit (Lambda=-0.25, c=0)

All covariance structures valid (AR(1), CS, UN), UN chosen, since best following AIC.

|  | | | | | | | | | | **95% Confidence interval (two-sided)** | |  |
| --- | --- | --- | --- | --- | --- | --- | --- | --- | --- | --- | --- | --- |
| **Analyte** | **Visit** | **Test** | **Reference** | **N_Test_** | **N_Reference_** | **LSMT** | **LSMR** | **LSM difference estimate** | **Standard Error** | **Lower** | **Upper** | **p-value** |
| IL10[pg/mL] | visit 3 | Bronchipret syrup | Control group | 13 | 8 | 0.6564263 | 0.5606151 | 1.2007019 | 0.1454 | 0.8858618 | 1.6686964 | 0.2320 |

| LSMs including confidence limits re-transformed | | |  |  |
| --- | --- | --- | --- | --- |
| **Analyte** | **ContrastsT** | | **F value** | **p-value (Pr > F)** |
| IL10[pg/mL] | Contrast Treatment at visit 3 | | 1.51 | 0.2320 |

| **Analyte** | **Effect** | **F value** | **p-value (Pr > F)** |
| --- | --- | --- | --- |
| IL10[pg/mL] | visit | 4.41 | 0.0277 |
|  | treatment | 2.52 | 0.1275 |
|  | treatment*visit | 2.47 | 0.1124 |
|  | baseline | 26.43 | <.0001 |

**IL6**

Box Cox Transformation required for model fit (Lambda=-0.75, c=0)

All covariance structures valid (AR(1), CS, UN), AR(1) chosen, since best following AIC.

|  | | | | | | | | | | **95% Confidence interval (two-sided)** | |  |
| --- | --- | --- | --- | --- | --- | --- | --- | --- | --- | --- | --- | --- |
| **Analyte** | **Visit** | **Test** | **Reference** | **N_Test_** | **N_Reference_** | **LSMT** | **LSMR** | **LSM difference estimate** | **Standard Error** | **Lower** | **Upper** | **p-value** |
| IL6[pg/mL] | visit 3 | Bronchipret syrup | Control group | 13 | 8 | 0.6638248 | 0.8004939 | 0.8036729 | 0.1388 | 0.6458192 | 1.0440911 | 0.0943 |

| LSMs including confidence limits re-transformed | | |  |  |
| --- | --- | --- | --- | --- |
| **Analyte** | **ContrastsT** | | **F value** | **p-value (Pr > F)** |
| IL6[pg/mL] | Contrast Treatment at visit 3 | | 2.93 | 0.0943 |

| **Analyte** | **Effect** | **F value** | **p-value (Pr > F)** |
| --- | --- | --- | --- |
| IL6[pg/mL] | visit | 0.44 | 0.6491 |
|  | treatment | 3.52 | 0.0762 |
|  | treatment*visit | 0.23 | 0.7929 |
|  | baseline | 7.13 | 0.0150 |

**Lymphocytes[/nl]**

No Box-Cox transformation required

All covariance structures valid (AR(1), CS, UN), CS chosen, since best following AIC.

|  | | | | | | | | | | **95% Confidence interval (two-sided)** | |  |
| --- | --- | --- | --- | --- | --- | --- | --- | --- | --- | --- | --- | --- |
| **Analyte** | **Visit** | **Test** | **Reference** | **N_Test_** | **N_Reference_** | **LSMT** | **LSMR** | **LSM difference estimate** | **Standard Error** | **Lower** | **Upper** | **p-value** |
| Lymphocytes[/nl] | visit 3 | Bronchipret syrup | Control group | 13 | 8 | 0.4009 | 0.4810 | -0.08015 | 0.1661 | -0.4189 | 0.2586 | 0.6328 |

| **Analyte** | **ContrastsT** | **F value** | **p-value (Pr > F)** |
| --- | --- | --- | --- |
| Lymphocytes[/nl] | Contrast Treatment at visit 3 | 0.23 | 0.6328 |

| **Analyte** | **Effect** | **F value** | **p-value (Pr > F)** |
| --- | --- | --- | --- |
| Lymphocytes[/nl] | visit | 3.01 | 0.0612 |
|  | treatment | 0.17 | 0.6888 |
|  | treatment*visit | 2.71 | 0.0796 |
|  | baseline | 1.21 | 0.2857 |

**Monocytes[/nl]**

No Box-Cox transformation required.

All covariance structures valid (AR(1), CS, UN), CS chosen, since best following AIC.

|  | | | | | | | | | | **95% Confidence interval (two-sided)** | |  |
| --- | --- | --- | --- | --- | --- | --- | --- | --- | --- | --- | --- | --- |
| **Analyte** | **Visit** | **Test** | **Reference** | **N_Test_** | **N_Reference_** | **LSMT** | **LSMR** | **LSM difference estimate** | **Standard Error** | **Lower** | **Upper** | **p-value** |
| Monocytes[/nl] | visit 3 | Bronchipret syrup | Control group | 13 | 8 | -0.1091 | -0.00273 | -0.1064 | 0.06397 | -0.2378 | 0.02503 | 0.1082 |

| **Analyte** | **ContrastsT** | **F value** | **p-value (Pr > F)** |
| --- | --- | --- | --- |
| Monocytes[/nl] | Contrast Treatment at visit 3 | 2.76 | 0.1082 |

| **Analyte** | **Effect** | **F value** | **p-value (Pr > F)** |
| --- | --- | --- | --- |
| Monocytes[/nl] | visit | 6.78 | 0.0030 |
|  | treatment | 1.71 | 0.2076 |
|  | treatment*visit | 0.86 | 0.4322 |
|  | baseline | 13.73 | 0.0016 |

**Neutrophils[/nl]**

Box Cox Transformation required for model fit (Lambda=-0.25, c=0)

All covariance structures valid (AR(1), CS, UN), AR(1) chosen, since best following AIC.

|  | | | | | | | | | | **95% Confidence interval (two-sided)** | |  |
| --- | --- | --- | --- | --- | --- | --- | --- | --- | --- | --- | --- | --- |
| **Analyte** | **Visit** | **Test** | **Reference** | **N_Test_** | **N_Reference_** | **LSMT** | **LSMR** | **LSM difference estimate** | **Standard Error** | **Lower** | **Upper** | **p-value** |
| Neutrophils[/nl] | visit 3 | Bronchipret syrup | Control group | 13 | 8 | 0.9923213 | 1.0168096 | 0.9760153 | 0.1156 | 0.7780466 | 1.2411495 | 0.8343 |

| LSMs including confidence limits re-transformed | |  |  |  |
| --- | --- | --- | --- | --- |
| **Analyte** | | **ContrastsT** | **F value** | **p-value (Pr > F)** |
| Neutrophils[/nl] | | Contrast Treatment at visit 3 | 0.04 | 0.8343 |

| **Analyte** | **Effect** | **F value** | **p-value (Pr > F)** |
| --- | --- | --- | --- |
| Neutrophils[/nl] | visit | 1.57 | 0.2224 |
|  | treatment | 0.00 | 0.9566 |
|  | treatment*visit | 1.21 | 0.3108 |
|  | baseline | 8.91 | 0.0080 |

**Platelets[/nl]**

Box Cox Transformation required for model fit (Lambda=-0.5, c=0)

All covariance structures valid (AR(1), CS, UN), UN chosen, since best following AIC.

|  | | | | | | | | | | **95% Confidence interval (two-sided)** | |  |
| --- | --- | --- | --- | --- | --- | --- | --- | --- | --- | --- | --- | --- |
| **Analyte** | **Visit** | **Test** | **Reference** | **N_Test_** | **N_Reference_** | **LSMT** | **LSMR** | **LSM difference estimate** | **Standard Error** | **Lower** | **Upper** | **p-value** |
| Platelets[/nl] | visit 3 | Bronchipret syrup | Control group | 13 | 8 | 4.8636195 | 3.31852 | 1.914573 | 0.8238 | 0.2704359 | 5.0504805 | 0.3636 |

| LSMs including confidence limits re-transformed | | |  |  |
| --- | --- | --- | --- | --- |
| **Analyte** | **ContrastsT** | | **F value** | **p-value (Pr > F)** |
| Platelets[/nl] | Contrast Treatment at visit 3 | | 0.87 | 0.3636 |

| **Analyte** | **Effect** | **F value** | **p-value (Pr > F)** |
| --- | --- | --- | --- |
| Platelets[/nl] | visit | 16.58 | <.0001 |
|  | treatment | 2.31 | 0.1456 |
|  | treatment*visit | 2.25 | 0.1346 |
|  | baseline | 0.10 | 0.7610 |

**TNF[pg/mL]**

No Box-Cox transformation required

All covariance structures valid (AR(1), CS, UN); AR(1) chosen, since best following AIC.

|  | | | | | | | | | | **95% Confidence interval (two-sided)** | |  |
| --- | --- | --- | --- | --- | --- | --- | --- | --- | --- | --- | --- | --- |
| **Analyte** | **Visit** | **Test** | **Reference** | **N_Test_** | **N_Reference_** | **LSMT** | **LSMR** | **LSM difference estimate** | **Standard Error** | **Lower** | **Upper** | **p-value** |
| TNF[pg/mL] | visit 3 | Bronchipret syrup | Control group | 13 | 8 | -5.1480 | -5.1742 | 0.02618 | 1.7225 | -3.5101 | 3.5625 | 0.9880 |

| **Analyte** | **ContrastsT** | **F value** | **p-value (Pr > F)** |
| --- | --- | --- | --- |
| TNF[pg/mL] | Contrast Treatment at visit 3 | 0.00 | 0.9880 |

| **Analyte** | **Effect** | **F value** | **p-value (Pr > F)** |
| --- | --- | --- | --- |
| TNF[pg/mL] | visit | 7.16 | 0.0023 |
|  | treatment | 0.07 | 0.7926 |
|  | treatment*visit | 0.22 | 0.7999 |
|  | baseline | 34.42 | <.0001 |
